# Study of a classification algorithm for AIEC identification in geographically distinct *E. coli* strains

**DOI:** 10.1038/s41598-020-64894-5

**Published:** 2020-05-15

**Authors:** Carla Camprubí-Font, Paula Bustamante, Roberto M. Vidal, Claire L. O’Brien, Nicolas Barnich, Margarita Martinez-Medina

**Affiliations:** 1 Laboratory of Molecular Microbiology, Department of Biology, Universitat de Girona, Girona, Spain; 2 Instituto de Ciencias Biomédicas, Facultad de Medicina, Universidad de Chile, Santiago, Chile; 3 Instituto Milenio de Inmunología e Inmunoterapia, Facultad de Medicina, Universidad de Chile, Santiago, Chile; 4 Graduate School of Medicine, University of Wollongong, Wollongong, NSW Australia; 5 Medical School, Australian National University, Canberra, Australian Capital Territory Australia; 6 Gastroenterology and Hepatology Unit, Canberra Hospital, Canberra, Australian Capital 14 Territory Australia; 7 UMR1071 Inserm/University Clermont Auvergne, INRAE USC2018, M2iSH, CRNH Auvergne, Clermont-Ferrand, France

**Keywords:** Microbiology, Inflammatory bowel disease

## Abstract

Adherent-invasive *Escherichia coli* (AIEC) have been extensively implicated in Crohn’s disease pathogenesis. Currently, AIEC is identified phenotypically, since no molecular marker specific for AIEC exists. An algorithm based on single nucleotide polymorphisms was previously presented as a potential molecular tool to classify AIEC/non-AIEC, with 84% accuracy on a collection of 50 strains isolated in Girona (Spain). Herein, our aim was to determine the accuracy of the tool using AIEC/non-AIEC isolates from different geographical origins and extraintestinal pathogenic *E. coli* (ExPEC) strains. The accuracy of the tool was significantly reduced (61%) when external AIEC/non-AIEC strains from France, Chile, Mallorca (Spain) and Australia (82 AIEC, 57 non-AIEC and 45 ExPEC strains in total) were included. However, the inclusion of only the ExPEC strains showed that the tool was fairly accurate at differentiating these two close pathotypes (84.6% sensitivity; 79% accuracy). Moreover, the accuracy was still high (81%) for those AIEC/non-AIEC strains isolated from Girona and Mallorca (N = 63); two collections obtained from independent studies but geographically close. Our findings indicate that the presented tool is not universal since it would be only applicable for strains from similar geographic origin and demonstrates the need to include strains from different origins to validate such tools.

## Introduction

The involvement of the adherent-invasive *Escherichia coli* (AIEC) pathotype in Crohn’s disease (CD) pathogenesis has been extensively supported, as many researchers have reported higher AIEC prevalence in CD patients than controls^1–9^, and mechanisms of pathogenicity have been linked with CD pathophysiology^10–17^. The ability to adhere to and invade intestinal epithelial cells, as well as, to survive and replicate inside macrophages are key characteristics of AIEC strains^2^. No gene or sequence exclusive to the AIEC pathotype has been identified, and AIEC identification currently remains challenging; the only way to identify an AIEC strain is by assessing bacterial infection in cell culture assays which are non-standardised and highly time-consuming^2^.

AIEC strains isolated to date are clonally diverse and belong to distinct serotypes. Although AIEC primarily fall into the B2 phylogroup, AIEC strains belonging to the A, B1, and D phylogroups have also been isolated^1,3,4,6,9,18–23^. In terms of virulence genes, AIEC resemble extraintestinal pathogenic *E. coli* (ExPEC), which are mostly non-invasive and the majority of them do not behave like AIEC^3,24–26^, with the exception of some isolates^26,27^.

Up to now, six genetic elements (*pduC, lpfA, lpfA* + *gipA, chuA*, 29 point mutations and 3 genomic regions) have been suggested as putative AIEC molecular markers^6,21,23,28,29^, however they either present low sensitivity or have been studied in a small number of strains. In a previous study conducted in our research group^30^, we designed a classification algorithm based on the identification of the nucleotides present in three Single Nucleotide Polymorphisms (SNPs). This algorithm displayed 82.1% specificity, 86.4% sensitivity and 84.0% accuracy within our Spanish strain collection. Given the high genotypic variability of AIEC, our aim was to validate the tool previously presented in AIEC/non-AIEC strains from distant geographical origins and ExPEC strains in order to assess the usefulness of these SNPs as molecular signatures for AIEC screening in external collections.

## Results

Confirmation of the validity of the algorithm^30^ in additional geographically distant AIEC/non-AIEC and ExPEC strains was performed.

**Figure 1:**
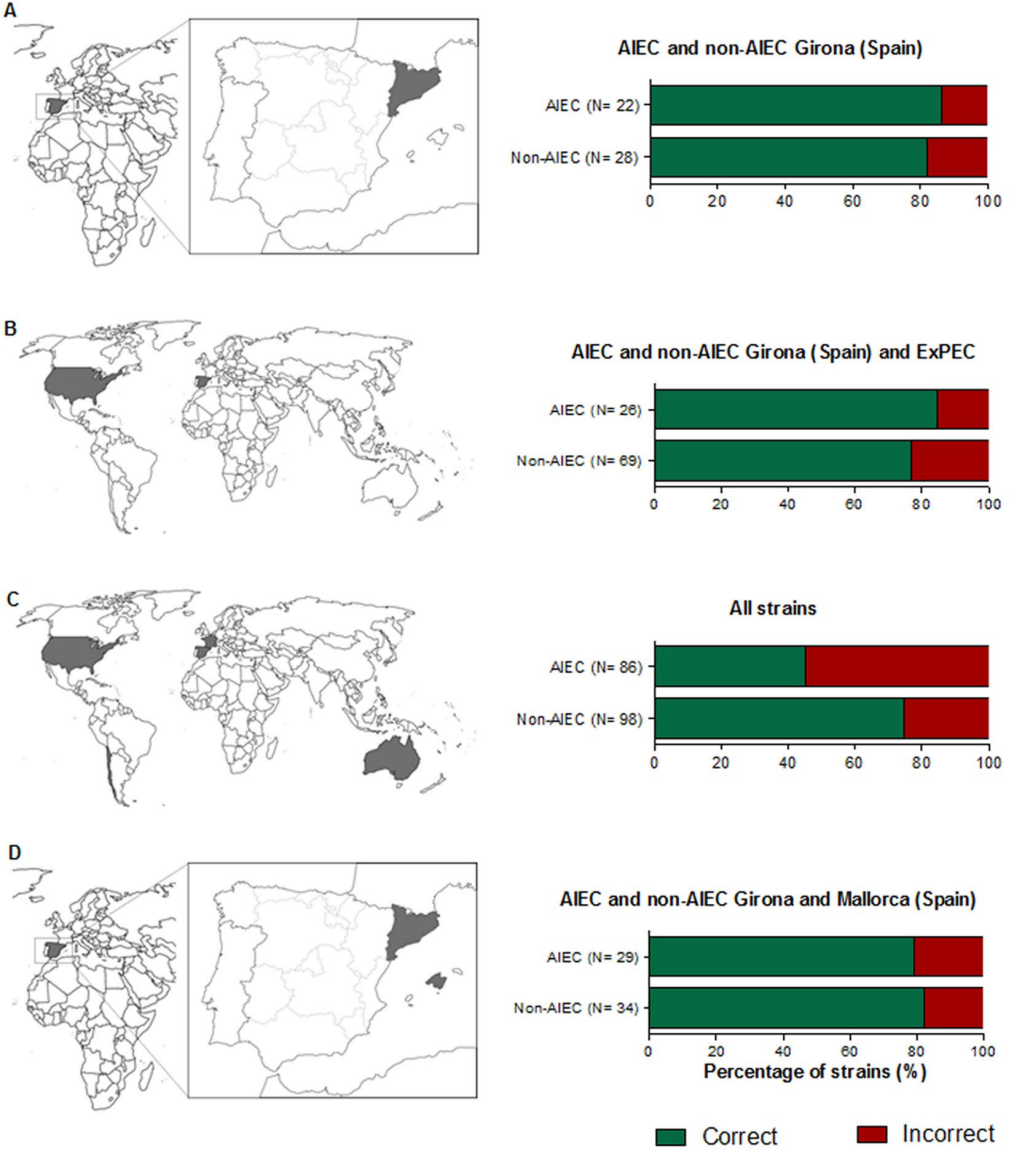
Geographical distribution of the isolates assessed in four groups of analysis and the percentage of strains that are correctly (green) or incorrectly (red) predicted by the SNP algorithm in comparison with their previous phenotypic characterisation. (**A**) AIEC/non-AIEC strains from Girona (Spain)^30^, including LF82 as a reference strain; (**B**) ExPEC (Spain and USA)^26,35,36^ and AIEC/non-AIEC strains from Girona (Spain)^30^; (**C**) AIEC/non-AIEC strains from Girona (Spain)^30^ and AIEC/non-AIEC from France, Chile^6^, Spain (Mallorca)^6^, Australia^33^ and ExPEC-Spain^26,36^ and ExPEC-America^35^; (**D**) AIEC/non-AIEC strains from Girona^30^ and Mallorca^6^ (Spain).

**Figure 2:**
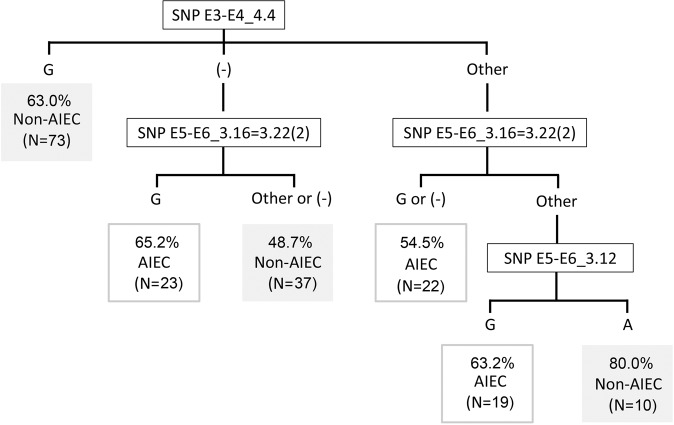
Classification algorithm for AIEC identification. Assessed in our collection and external strain collections (France, Chile^6^, Spain (Mallorca)^6^, Australia^33^ and ExPEC-Spain^26,36^ and ExPEC-America^35^). Percentages represent the proportion of strains that are correctly predicted as AIEC or non-AIEC based on the result for each SNP combination. The number of total strains corresponding to each condition is indicated. (−): no amplification; other: a nucleotide different from guanine (G) or overlapping peaks.

When all AIEC/non-AIEC strains from Girona, Mallorca, France, Chile and Australia, as well as ExPEC strains were analysed, 73/98 of the non-AIEC strains were correctly classified but only 39/86 of the AIEC strains were appropriately predicted, resulting in a high probability of obtaining false negatives (54.6%). Therefore, in comparison to the values obtained within our strain collection (82.1% specificity, 86.4% sensitivity and 84.0% accuracy), the global accuracy was significantly reduced (60.9%), with decreased specificity (74.5%) and especially lower sensitivity (45.4%) (Table 1, Fig. 1). In contrast to the previous study^30^, the SNPs that were found to be differentially distributed among our AIEC and non-AIEC strains (E3-E4_4.4 and E5-E6_3.16 = 3.22(2)) showed similar frequencies according to phenotype when all the strains were considered (Table 2). According to the algorithm^30^, strains displaying guanine (G) in SNP E3-E4_4.4 are classified as non-AIEC, and the same occurs for those that do not have the gene (−) where SNP E3-E4_4.4 is located and display a nucleotide other than G at SNP E5-E6_3.16 = 3.22(2). Indeed, most AIEC strains (54.6%) were incorrectly classified because they accomplished these conditions (Fig. 2). Other possible SNP combinations were considered for all the strains included in the study but none improved the precision of the algorithm.

**Table 1:** Summary table of the accuracy of the tool in each strain collection analysed.

	Observed	Predicted		Predictive Values	
		Non-AIEC	AIEC	% Correct	Accuracy
AIEC/non-AIEC Spain (Girona)^30^	Non-AIEC	23	5	82.1	84.0
	AIEC^#^	3	19	86.4	
AIEC/non-AIEC Spain (Mallorca)^6^	Non-AIEC	5	1	83.3	69.2
	AIEC	4	3	57.1	
AIEC/non-AIEC France	Non-AIEC	0	0	0	32.3
	AIEC	23	11	32.3	
AIEC/non-AIEC Chile^6^	Non-AIEC	3	0	100	33.3
	AIEC	6	0	0	
AIEC/non-AIEC Australia^33^	Non-AIEC	12	8	60.0	42.4
	AIEC	11	2	15.4	
ExPEC Spain and USA^26,35,36^	Non-AIEC	30	11	73.2	73.3
	AIEC	1	3	75.0	
AIEC/non-AIEC Spain (Girona) and ExPEC	Non-AIEC	53	16	76.8	78.9
	AIEC	4	22	84.6	
AIEC/non-AIEC Spain (Girona) and AIEC/non-AIEC Spain (Mallorca)	Non-AIEC	28	6	82.3	80.9
	AIEC	6	23	79.3	
All strains*	Non-AIEC	73	25	74.5	60.9
	AIEC	47	39	45.4	

^#^Include LF82 as AIEC reference strain. *Include AIEC/non-AIEC strains from Girona, Mallorca, France, Chile and Australia, as well as, ExPEC strains from Spain and USA.

**Table 2:** Frequency of particular nucleotide variants in SNP E3-E4_4.4 and E5-E6_3.16 = 3.22(2) with respect to phenotype in two collections of AIEC/non-AIEC strains. Values are given in percentages with respect to the total number of AIEC or non-AIEC strains.

SNP	Variant	Girona strain collection^30^			AIEC/non-AIEC from diferent geographic origins and ExPEC strains.		
		AIEC (N = 22)	Non-AIEC (N = 28)	p-value	AIEC (N = 86)	Non-AIEC (N = 98)	p-value
E3-E4_4.4	G	9.1	42.9	0.010	31.8	46.5	0.246
	A	13.6	7.1		9.4	8.1	
	R	55.6	21.43		21.2	17.2	
	(−)	21.7	28.6		37.6	28.3	
E5-E6_3.16 = 3.22(2)	G	31.8	3.6	0.012	24.7	14.9	0.237
	C	13.6	39.3		17.6	22.3	
	Others*	54.6	57.1		57.6	62.8	

*Others include those strains having T, S, K, Y or not having the gene where the SNP is encompassed.

Despite global accuracy of the algorithm being much lower when all strains were considered, the method was suitable for geographically close strain collections. Indeed, if only Spanish strains (Girona and Mallorca) (N = 63) were considered, the accuracy of the tool was maintained (80.9%) (Table 1). Specificity was also good (82.3%), meaning there was a low probability of false positives (17.7%) (Fig. 1). Therefore, strains from different laboratory collections, but of similar geographical origin, were suitable for screening by this method.

The inclusion of ExPEC strains (N = 45) revealed that the tool was also useful for distinguishing the ExPEC and AIEC pathotypes, since 84.6% of strains displaying the AIEC phenotype were correctly classified, with a global accuracy of 78.9% (Table 1, Fig. 1).

These results demonstrated that the classification algorithm presented has limited applicability for all *E. coli* strains assessed. However, this novel molecular tool showed promising results for Spanish AIEC and ExPEC strains.

## Discussion

The identification of molecular tools or rapid tests to easily identify the AIEC pathotype would be of great interest to scientists studying the epidemiology of the pathotype, as well as clinicians hoping to detect which patients are colonised by AIEC to apply personalised treatments. Although several studies have been conducted with this aim in mind, there is still no molecular signature specific to AIEC^6,21,23,28,29^.

In a previous study we performed comparative genomics of three AIEC/non-AIEC clone pairs and presented a classification algorithm that combines three SNPs, allowing for the classification of phylogenetically and phenotypically diverse *E. coli* isolates with a high accuracy rate in our strain collection^30^. Since the application of a molecular tool could assist in overcoming the problem of AIEC identification, we further tested the specificity and sensitivity of the tool in additional geographically distant and phylogenetically diverse AIEC strains, as well as ExPEC strains, which share genetic and phenotypic features^3,24–26^.

The tool was found to be accurate enough to distinguish between AIEC and ExPEC strains, since the sensitivity was 84.6% and the accuracy was 78.9%. In this case, we assessed both AIEC/non-AIEC from Girona (Spain) and ExPEC strains, the latter being mostly Spanish isolates. These results indicated that for a given geographic origin this algorithm could be applied to differentiate ExPEC from AIEC. So far, most of the studies looking for AIEC biomarkers have not included ExPEC strains in their analysis^6,21,23,28^. There is only one that focused on synonymous and non-synonymous SNPs along the genome of four B2-AIEC strains that could differentiate them from other B2-non-AIEC and B2-ExPEC genomes available in databases. Although they found 29 SNPs that could separate AIEC from non-AIEC using a bioinformatics approach, but did not include the three SNPs in the presented algorithm, it did not find a signature sequence that distinguishes AIEC from ExPEC^29^. It is not possible to determine whether the high accuracy value we reported is due to similar geographic origin (40 from Spain and 5 USA) or not. Thus the inclusion of other ExPEC strains would be needed to validate the tool further. Unfortunately, the predicted values of the tool decreased considerably (60.9% of accuracy) when strains across several geographic regions were considered. AIEC isolates from France, Chile and Australia were poorly discriminated with the SNP algorithm presented, resulting in significantly reduced sensitivity values (32.3, 0 and 15.4% respectively). Of note, this algorithm may be suitable for Spanish strains, because the accuracy was still high when two different collections of strains were studied (Girona and Mallorca) (80.9% accuracy). Taking into account that the variable gene content of *E. coli* is highly variable across different geographic regions^31^, this variation contributes to the algorithm not being applicable across geographically diverse regions and it is subjected to possible variations in the accuracy presented in a particular country.

In conclusion, the molecular tool that we previously proposed^30^ is not universal since its accuracy was reduced to 60.9% once a larger strain collection from different geographic locations and pathotypes was screened. We suspect it might be a good discrimination tool for a particular geographic location, in this case Spain. However, this observation should be confirmed with the addition of other Spanish strain collections including AIEC, non-AIEC, and other *E. coli* intestinal and extraintestinal pathotypes. The study of new SNPs that could be useful to distinguish between AIEC/non-AIEC strains from different geographical origins might be time-consuming and unprofitable and should consider many aspects that make it even more complicated (for example, the moment of strain isolation and the patient’s treatment). Therefore, we believe that new approaches (e.g. transcriptomics, metabolomics or epigenetics) should be applied to find a universal AIEC biomarker that could be used as a rapid standardised method for detecting AIEC from *E. coli* isolates, or maybe just *E. coli* isolates that have a strong colonizing ability. Nonetheless, there is a possibility that a no universal marker exists and then it would be interesting to look for a biomarker that englobes the majority of AIEC strains^32^. In any case, this work highlighted the importance of validating putative molecular markers in a diverse strain collection, in terms of geographic origin and pathotype, in order to assess whether or not it could be used universally.

## Methods

The SNPs included in the algorithm (E3-E4_4.4, E5-E6_3.16 = 3.22(2) and E5-E6_3.12) were screened by PCR and Sanger sequencing. Primers and PCR conditions are indicated in Table 3. Apart from the strains assessed in the previous study (22 AIEC and 28 non-AIEC, which includes LF82 strain)^30^, this collection comprised 60 AIEC and 29 non-AIEC strains mainly isolated from CD patients and controls from distinct geographical origin (Spain (Mallorca)^6^, Chile^6^, France and Australia^33^) (Table S1). Most of these strains were phenotypically characterised in previous studies^6,33^. The adhesion and invasion indices of 25/33 Australian strains were measured in this study as previously described^1,30,34^ in order to classify them phenotypically as AIEC or non-AIEC. In addition, 45 strains isolated from patients with extraintestinal diseases were also included; these were previously isolated from American patients with meningitis^35^, and Spanish patients with sepsis^26^ or urinary tract infection^36^ (Table S1). Phenotypic characterisation of these strains was performed by Martinez-Medina *et al*.^26^; in which four strains presented the AIEC-phenotype and were considered as such in the analysis and 41 did not (these were classified as non-AIEC).

**Table 3:** Primers and PCR conditions used to amplify fragments of the genes in which the Confirmed SNPs were located.

Gene ID	Primer Forward (5′ to 3′)	Primer Reverse(5′ to 3′)	Annealing temperature (°C)
E3-E4_4.4	ATATTCAGCCTGTCCGCAAT	CGCATCATCACTTCCATCTG*	57
E5-E6_3.12	GAAAAAGTCGCCCATGAGAC*	CGCAACACCAGAGGGTTAAT	57
E5-E6_3.16 = 3.22	GCTGAACCATTCATTCACG*	TTATTGCAGAAAAGCGAGAGG	54

PCR program: 1 cycle at 95 °C for 5 min, 30 cycles of 15 sec at 95 °C and 45 sec at the primer annealing temperature, finally, one cycle at 72 °C during 10 min. All primers were used at 0.2 μM; PCR Buffer II at 1×; MgCl_2_ at 1.5 mM; dNTPs at 200 µM and AmpliTaq Gold polymerase 1.25 units/reaction. *Indicate the primer used for sequencing.

Strains studied in this study were previously isolated under the approval of the Ethics Committee 183 of Clinical Investigation of the Hospital Josep Trueta of Girona on May 22 2006; Ethical 184 committee of Hospital Saint-Louis (CPP#2009/17); Institutional Review Board of Clínica Las 185 Condes, Faculty of Medicine, Universidad de Chile; Ethics Committee of the Northern 186 Metropolitan Health Service, Santiago, Chile; the Balearic Islands’ Ethical Committee, Spain; and 187 ACT Health Human Research Ethics Committee (ETH.5.07.464). Subjects gave written 188 informed consent in accordance with the Declaration of Helsinki.

The differences in the distribution of nucleotides present in each polymorphic site between phenotype were calculated using the Χ^2^ test. To establish the usefulness of the algorithm for AIEC identification, the specificity, sensitivity and accuracy values were measured as follows: Sensitivity (%)= (true positives/(true positives + false negatives)) × 100, Specificity (%)= (true negatives/(true negatives + false positives)) x 100; and, Accuracy (%)= ((true positives + true negatives)/(total of cases)) × 100. A p-value ≤ 0.05 was considered statistically significant in all cases.

## Supplementary information

Supplementary information.
